# Integrating multiomics and modern breeding tools for accelerating genetic improvement in Annonas

**DOI:** 10.1007/s10142-025-01653-7

**Published:** 2025-07-12

**Authors:** Shri Hari Prasad, Grant Bignell, Rhys G.R. Copeland, Vanika Garg, Annapurna Chitikineni, Robert J. Henry, Natalie Dillon, Reyazul Rouf Mir, Rajeev K. Varshney

**Affiliations:** 1 WA State Agricultural Biotechnology Centre, Centre for Crop and Food Innovation, Food Futures Institute, Murdoch University, Murdoch, WA 6150 Australia; 2 Maroochy Research Facility, Queensland Department of Primary Industries, Nambour, QLD 4560 Australia; 3 Queensland Alliance for Agriculture and Food Innovation (QAAFI), The University of Queensland, St Lucia, QLD 4072 Australia; 4 Queensland Department of Primary Industries, Mareeba, QLD 4880 Australia; 5 Division of Genetics & Plant Breeding, Faculty of Agriculture (FoA), SKUAST-Kashmir, Wadura Campus, Sopore, 193201 Kashmir, J&K India

**Keywords:** *Annona*, Annonaceae, Custard apple, Genomics, Multiomics, Crop improvement

## Abstract

Custard apples (*Annona* spp.) are among the most important horticultural crops in the world, including Australia. The genus *Annona* comprises several economically and nutritionally significant species, including atemoya, cherimoya, sugar apple, ilama, soursop, bullock’s heart, and bibra. These fruits are valued for their exotic taste and are popular backyard fruit crops in many countries. While some species are commercially cultivated and exported, the broader potential of these crops remains largely untapped. Despite their historical significance, these *Annona* species remain neglected or underutilised, with breeding efforts restricted to only a few countries. Extensive genetic resources, including germplasm collections, candidate genotypes, and mapping populations, are available for crop improvement. Traditional breeding methods - such as selection, crossbreeding, and mutation breeding – have been widely applied alongside modern breeding approaches like marker-assisted selection (MAS). However, several challenges, such as a lack of information regarding the crop and a long juvenile period, hinder crop improvement in custard apples. Recent advancements and affordability of sequencing technologies have enabled an increase in the number of multiomics studies, especially genomics and transcriptomics within *Annona* species. Integrating these data with proteomics, metabolomics, and phenomics will facilitate the genetic dissection of important traits in *Annona*. This review provides a comprehensive overview of the current advancements and future prospects of multiomics tools and technologies developed and their potential to accelerate custard apple breeding programs.

## Introduction

*Annona* L. is the most important genus in the family Annonaceae (commonly known as the custard apple family). It comprises approximately 160 species (Chatrou et al. 2012; Serbin et al. 2023), including a diverse array of flowering plants, which hold significant economic and ecological importance (Maas et al. 2019). While *Annona* includes a diverse array of species, only a few produce commercially valuable edible fruits. These include cherimoya (*A. cherimola*), valued for its delicate flavour and creamy texture (Lora et al. 2018); sugar apple (*A. squamosa*), popular for its sweet taste and unique appearance (Padmanabhan and Paliyath 2016); hybrid atemoya (*A.* × *atemoya*), a luscious cross between cherimoya and sugar apple (Lim 2012a); bullock’s heart (*A. reticulata*), characterised by its heart-shaped fruits (Niu et al. 2020); soursop (*A. muricata*), known for its large spiny fruits with tangy flavour (Padmanabhan and Paliyath 2016); ilama (*A. diversifolia*), noted for its distinctive leaf bracts and varied fruit colours (Lim 2012b); and biriba (*A. mucosa*), characterised by its yellow fruits with wart-like protuberances (Lim 2012c).

**Fig. 1:**
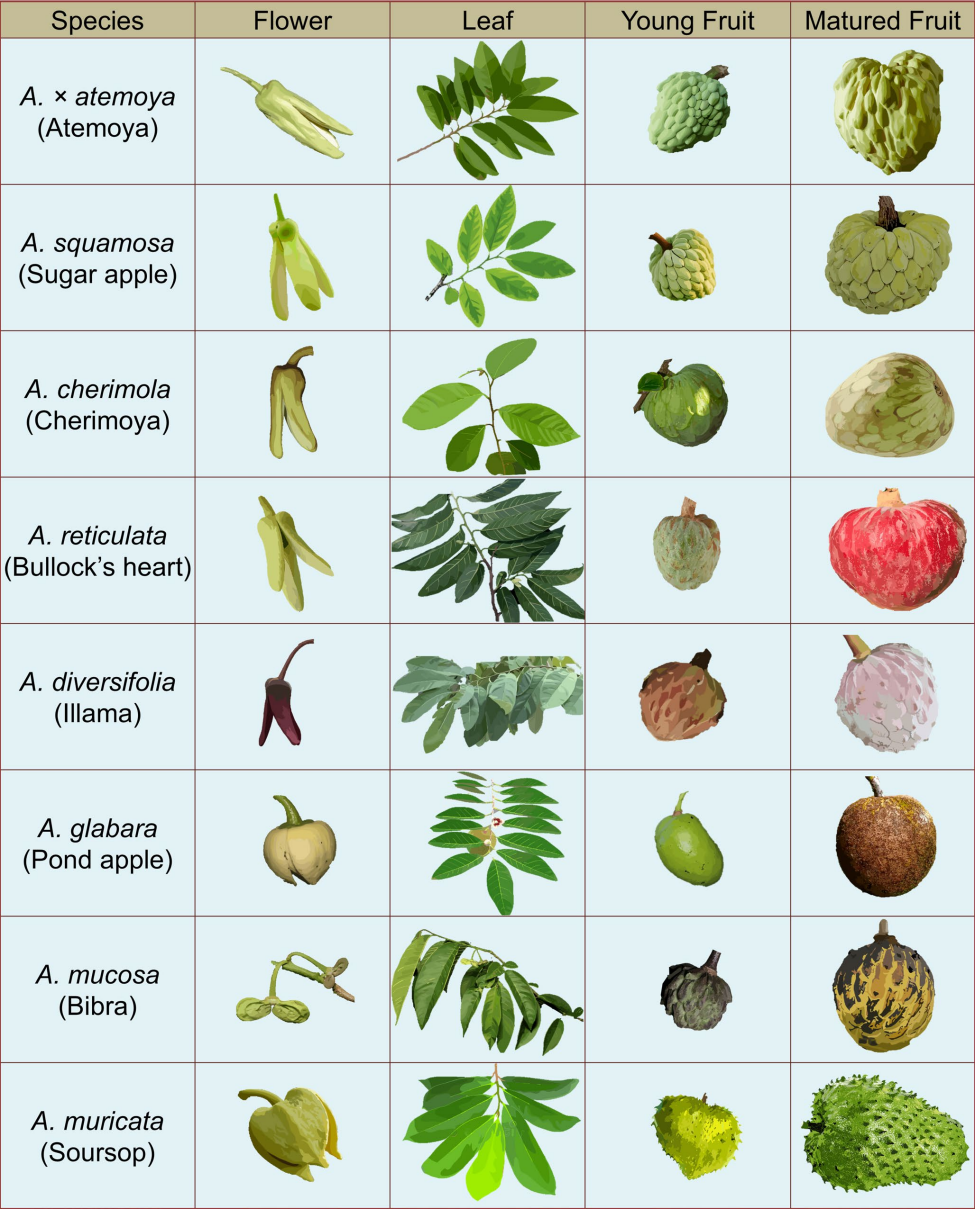
Morphological diversity within the genus *Annona*, displaying variations in flowers, leaves, young fruits, and mature fruits across important edible species

*Annona* species exhibit diverse growth habits, and display a remarkable array of fruit shapes, sizes, and flavours (Fig. 1). Cytogenetically, *Annona* species are largely diploid (2*n* = 14) (Martin et al. 2019), though polyploidy has been reported in some species. For instance, *A. glabra*,* A. lutescens*,* A. neolaurifolia* and *A. exsucca* are tetraploid, while *A. mucosa*, *A. pulchrinervis* and *A. neosalicifolia* are hexaploid (Martin et al. 2019; Serbin et al. 2023). The genome size of *Annona* species ranges from 587 Mb to 1137 Mb (Talavera et al. 2023; Peña-Ramírez et al. 2024).

*Annona* species are cultivated both as backyard crops and commercially for export in many countries. *Annona* fruits are highly nutritious, nutritional analyses of various *Annona* species, including sugar apple (Brandão and Santos 2016; David et al. 2019), atemoya (Brandão and Santos 2016), cherimoya (Albuquerque et al. 2016), soursop (David et al. 2019), and bullock hearts (David et al. 2019), have revealed that they are rich sources of total sugars, ascorbic acid, thiamine, riboflavin, and niacin. They also contain significant amounts of minerals such as calcium, magnesium, and phosphorus, along with high levels of antioxidants (de Moraes et al. 2020). Despite their nutritional value and market potential, *Annona* species remain underutilised, due to production challenges such as low productivity, small fruit size, susceptibility to biotic and abiotic stresses, delicate nature of fruit, shelf life, and the impact of climate change have significantly constrained their production. Overcoming these constraints and meeting increasing consumer demand for improved fruits necessitates innovative breeding strategies focused on developing climate-resilient, nutritionally superior, and flavourful cultivars.

**Fig. 2:**
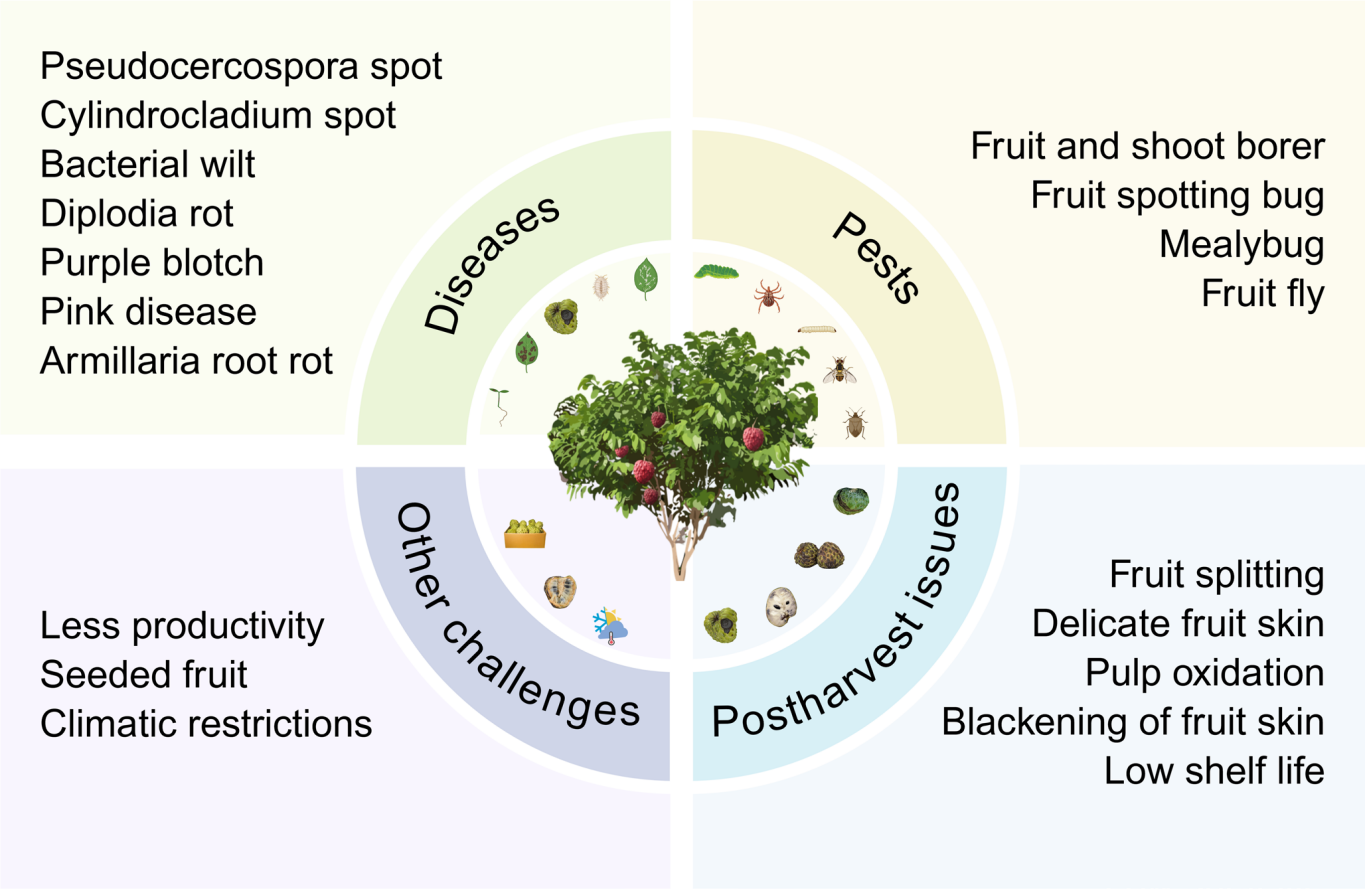
Key challenges in *Annona* production: diseases, pests, postharvest issues, and other factors all contribute to reduced productivity and quality. Created with Biorender.com

Traditional breeding methods have proven insufficient to meet the growing demand for improved cultivars with enhanced yield, quality, and stress tolerance. Advances in next-generation sequencing (NGS) technologies have enabled the rapid and cost-effective generation of multiomics data. Integrating these datasets with trait-specific information can provide deeper insights into the genetic basis of important traits, enabling genomics-assisted breeding (Varshney et al. 2005). Efforts to utilise these cutting-edge technologies in crop improvement are in progress for many fruit crops (Iwata et al. 2016; Lee et al. 2024). However, the development and implementation of such sophisticated omics technologies in *Annona* is still in its infancy.

A wealth of genetic resources encompassing numerous elite selections, intra- and interspecific hybrid populations, mutant lines, and diverse wild genotypes are available for *Annona* breeding. The genomic resources are steadily increasing, with multiple genome assemblies available across different species and numerous transcriptomic, proteomic and metabolomic studies conducted to date. Rich genetic diversity has been elucidated through numerous marker studies; however, research on trait-associated markers remains limited.

This review provides a comprehensive overview of *Annona* breeding, critically evaluating the limitations of conventional breeding approaches and exploring the transformative potential of multiomics technologies. We discuss the available genetic resources, molecular marker studies, genetic maps, QTL analyses, and multiomics research. Additionally, we explore modern breeding strategies, such as genomics-assisted breeding, to fast-track *Annona* breeding programs.

## Challenges facing Annonas

Despite the increasing global demand for *Annona* fruits, their commercial cultivation, post-harvest management and market reach are fraught with considerable challenges. These issues include biotic stress, including pests and diseases, abiotic stress, inherent fruit characteristics and environmental limitations (Fig. 2). Seed and fruit borers, fruit flies, mealybugs and fruit spotting bugs are the major insect pests that pose a serious threat to *Annona* species and cause substantial losses (George et al. 2015; Shylesha and Mani 2022; Romero et al. 2025). However, most of these studies are on economically relevant pests that affect commercially important *Annona* species (Romero et al. 2025), meaning their impact on non-commercial and wild species is likely underestimated and is a concern. Complementing the damages caused by pests, numerous bacterial and fungal diseases also threaten *Annona* cultivation and production. These diseases include fruit and leaf spots (*Cylindrocladium*, *Pseudocercospora*), Anthracnose (*Colletotrichum gloeosporioides*), Purple Blotch (*Phytophthora palmivora*), Pink Disease (*Corticum salmonicolor*), Armillaria Root Rot (*Armillaria luteobubalina*) and Bacterial Wilt (*Ralstonia solanacearum*) (George et al. 2015; Sharma and Lakpale 2020). A significant concern is how altered and erratic climatic conditions can influence the pest and disease dynamics, potentially leading to minor pests and diseases emerging as major ones. Among the abiotic stresses, drought, flooding, heat, cold, and salinity stress are the most prevalent (Leal and Paul 2023). As tolerance to these stresses varies among species, these stresses affect plant growth, metabolism, fruit quality, and fruit yield. Beyond the biotic and abiotic stressors, postharvest challenges like fruit splitting, delicate fruit skin, low shelf life, pulp oxidation, and blackening of fruit skin further limit market availability and consumer preference. Furthermore, there is a market desire for seedless cultivars and constant demand for increased productivity, but breeding and cultivation are hindered by climatic factors.

## Genetic resources: from wild relatives to elite cultivars

**Fig. 3:**
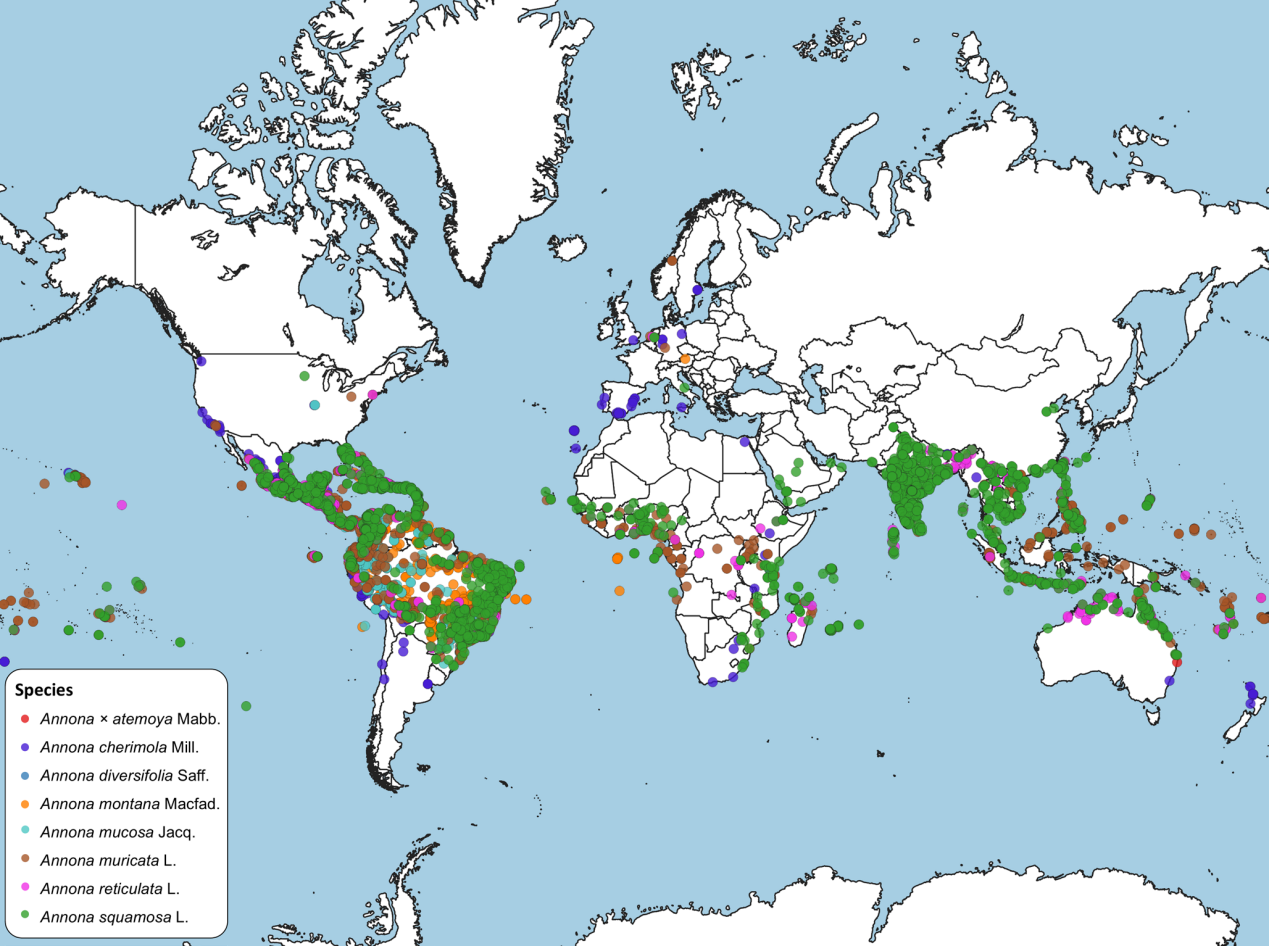
Global distribution of key *Annona* species based on 20,412 georeferenced entries obtained from Global Biodiversity Information Facility (GBIF; https://www.gbif.org/). Each dot represents an individual *Annona* specimen record. Map compiled using QGIS v3.42.3

While the genus *Annona* is highly diverse, germplasm conservation efforts primarily focus on commercially important species such as *A. cherimola*, *A. muricata*, *A. squamosa*, and, to a lesser extent, *A.* × *atemoya*. Currently, approximately 1,741 accessions representing 12 *Annona* species, including the interspecific hybrid atemoya, are preserved as ex situ collections around the world. These are distributed across 67 institutional collections across 34 countries (Sushmitha and Sakthivel 2023). Cherimoya (*A. cherimola*) germplasm collections are maintained in Mexico, Peru, Ecuador, and Spain (Larranaga et al. 2024). Diverse sugar apple (*A. squamosa*) accessions exist in India, Brazil and Thailand, while Australia, Thailand, India, China and the USA hold diverse atemoya (*A.* × *atemoya*) accessions (George et al. 2010). Additionally, important soursop (*A. muricata*) collections are found in Brazil, Sri Lanka, and China. A map illustrating the global distribution of some key *Annona* species is provided in Fig. 3. Despite these efforts, the conservation of other *Annona* species like *A. diversifolia*, *A. reticulata*, and *A. mucosa*, which hold potential commercial value and other wild species, which could serve as vital sources of important traits like resistance to biotic and abiotic factors, remains limited. Expanding germplasm preservation initiatives is essential for safeguarding genetic diversity and facilitating future crop improvement.

The presence of *Annona* species adapted to subtropical and tropical climates (Escobedo-López et al. 2019), along with their graft compatibility, offers a valuable opportunity to utilise wild relatives as gene sources or rootstocks for enhanced adaptability. While various conservation efforts exist to a smaller extent, an efficient conservation effort requires a multipronged approach. For in situ conservation efforts, it is crucial to educate the communities and support efforts to promote on-farm conservation and also identify and conserve regions of high natural diversity (Lora et al. 2018; Sushmitha and Sakthivel 2023). For ex situ conservation strategies, studies to determine the viability of seeds after short, medium, and long-term storage are lacking. While there are studies detailing methods for in vitro micropropagation and regeneration (Encina et al. 2014), a comprehensive germplasm collection using these methods has not been established. Field genebanks, often part of ongoing breeding efforts, are present, but they typically host less diversity. Such collections are more often short-lived due to land, labour and funding constraints for maintaining them. Furthermore, along with in situ germplasm, they are susceptible to abiotic, biotic and natural hazards (Sushmitha and Sakthivel 2023). However, a critical gap remains in the identification of diverse regions for optimal germplasm collection, establishing effective methods for comprehensive germplasm conservation, and facilitating the efficient utilisation and exchange of these resources. The availability and application of genomic resources, such as genomes and pangenomes, will significantly enhance our understanding of the genetic potential of various *Annona* species. These resources are vital in mapping the regions of genetic diversity and identifying the hotspots and vulnerable areas. Genomic resources have provided key insights, for example, a genetic diversity analysis of 1,765 cherimoya accessions, combined with climate modelling, revealed that the Central American region is likely the primary centre of origin for cherimoya, with a secondary distribution to the Andean region (Larranaga et al. 2017). While this contradicts the previously believed South American origin, such findings are pivotal for redirecting conservation efforts and also for performing climate impact analysis to identify vulnerable areas and implement targeted conservation practices. Furthermore, by exploring population diversity and understanding the genetic basis of various important traits, efforts can be made to facilitate effective utilisation of these resources in future crop improvement initiatives. Previous studies have characterised *Annona* species for several important morphological traits (Pinto et al. 2005; Andrés-Agustín et al. 2006; Awachare et al. 2018; Moreira-Macías et al. 2020; Jnapika et al. 2021; Larranaga et al. 2024), leading to the development of several high-value cultivars. The variability in fruit and tree characteristics across different species has been exploited in many breeding programs due to their relatively easy hybridisation (Zill and Mahdeem 1998; Jalikop 2010). This has resulted in the development of numerous elite cultivars. Moreover, many *Annona-*growing countries possess species with varying degrees of domestication, including semi-cultivated and wild species, further enriching the gene pool (Escobedo-López et al. 2019). In addition, natural and human selection under diverse climatic conditions has resulted in a wide variety of locally adapted landraces, some well-known for their fruit characteristics and are usually named based on colour, origin, or even specific plant parts (Sushmitha and Sakthivel 2023). Additionally, atemoya mutant lines developed through gamma irradiation (George et al. 2005, 2010) and colchicine treatment (George et al. 2002, 2010) to develop seedless cultivars are available. Furthermore, naturally occurring seedless sugar apple mutants have also been identified (Lora et al. 2011). Further screening of semi-domesticated and wild germplasms may uncover additional valuable natural mutants.

## Beyond morphology: development and use of molecular markers

The Annonaceae family exhibits significant intra- and interspecific variation due to its highly cross-pollinated nature. The establishment of plantations can be attributed to the proliferation of seedlings that have naturally emerged through the dispersal of seeds; consequently, they exhibit significant diversity. Historically, morphological characteristics have been employed to evaluate the untapped potential of germplasm for the development of high-yielding varieties with improved fruit quality (Folorunso and Modupe 2007). However, conventional morphological markers are subject to considerable effects from edaphic and climatic factors, which raises concerns about their reliability due to the strong environmental impact they experience (Kumar et al. 2014). Consequently, molecular markers provide a more robust and precise approach for assessing genetic diversity.

Over the years, researchers have used several types of molecular markers to assess genetic diversity (Table 1). Isozyme analysis was initially used to study the genetic diversity of *Annona* spp. in the 1980s (Ellstrand and Lee 1987; Pascual et al. 1993; Perfectti and Pascual 1998a, 2004, 2005). However, isozyme technology is limited by the low number of markers it provides. To overcome this limitation, DNA-based markers, such as simple sequence repeat (SSR) or microsatellite, amplified fragment length polymorphism (AFLP), random amplified polymorphic DNA (RAPD), and inter simple sequence repeat (ISSR) markers, have been used to assess genetic diversity.

**Table 1:** Summary of key molecular marker studies in *Annona* spp

Marker system	Species	No. of polymorphic primers/bands/loci	Individuals	Purpose	Reference
Isozyme	*A. cherimola* *A.* × *atemoya*	15	16	Varietal identification	Ellstrand and Lee (1987)
	*A. cherimola*	13	7	Germplasm characterisation	Pascual et al. (1993)
	*A. cherimola* *A.* × *atemoya*	15	210	Germplasm characterisation	Perfectti and Pascual (1998a)
	*A. cherimola* *A. atemoya*	13	210	Genetic diversity	Perfectti et al. (2004, 2005)
RAPD	*A. cherimola* *A. squamosa* *A.* × *atemoya* F_1_ population of Jete × Champa	52	6 + 38	Fingerprinting genotypes within and between *Annona* species	Ronning et al. (1995)
	*A. muricata*	14	9	Genetic relationship	Brown et al. (2003)
	*A. squamosa*	19	11	Genetic diversity	Bharad et al. (2008)
	*Annona* spp.	25	-	Genetic diversity	Ahmad et al. (2010)
	*A. squamosa*	48	64	Genetic diversity	Guimarães et al. (2013)
	*A. muricata*	151	70	Genetic diversity	Suratman et al. (2015)
	*A. cherimola* *A. squamosa* *A.* × *atemoya* *A. muricata* *A. reticulata*	98	20	Genetic diversity	Anuragi et al. (2016)
	*A. muricata*	59	42	Genetic diversity	Brisibe et al. (2017)
AFLP	*A. cherimola*	88	19	Genetic diversity	Rehman et al. (1998)
	*A. squamosa*	8	2	To distinguish two sugar apple cultivars, Nua and Nang	Thanananta and Thanananta (2008)
	*A. squamosa*	-	7	Genetic diversity	Zhichang et al. (2011)
	*A. crassiflora*	-	106	Genetic diversity	Egydio-Brandão et al. (2016)
SSR	*A. cherimola* *A. x atemoya* *A. squamosa* *A. montana* *A. glabra* *R. emarginata* *R. salicifolia*	15	23	Characterisation and cross-species amplification	Escribano et al. (2004)
	*A.* × *atemoya* *A. cherimola* *A. squamosa* *A. reticulata* *A. diversifolia* *R. deliciosa*	-	27	Fingerprinting the genotypes used in the Australian custard apple breeding programme.	George et al. (2008)
	*A. cherimola*	14	279	DNA fingerprinting	Escribano et al. (2007)
	*A. senegalensis*	3	135	Genetic diversity	Kwapata et al. (2007)
	*A. cherimola*	52	23	DNA fingerprinting and genetic diversity	Escribano et al. (2008)
	*A. crassiflora*	10	32	Genetic diversity	Pereira et al. (2008)
	*A. cherimola*	-	1504	Genetic diversity	Van Zonneveld et al. (2012)
	*A. cherimola* *A. squamosa* *A.* × *atemoya* *A. muricata* *A. reticulata*	37	20	Genetic diversity	Anuragi et al. (2016)
	*A. squamosa*	3	-	Genetic diversity	Rodrigues et al. (2024)
ISSR	*Annona* spp.	18	-	Genetic diversity	Ahmad et al. (2010)
	*A. squamosa*	101	19	Genetic diversity	Sá et al. (2022)

Abbreviations: AFLP = amplified fragment length polymorphism, RAPD = randomly amplified polymorphic DNA, SNP = single nucleotide polymorphisms, SSR = simple sequence repeat, ISSR = Inter simple sequence repeat

The improved efficacy and cost-effectiveness of sequencing technologies have led to an increase in the demand for high-throughput technologies, enabling researchers to employ high-density markers spanning the entire genome to evaluate genetic diversity. The identification of genes associated with particular traits has also enabled the creation of trait-specific markers. A recent study explored the effective use of diversity array technology (DArT) markers, namely, dominant silicoDArT markers and codominant single nucleotide polymorphism (SNP) markers, for the analysis of *Annona* accessions. In this study a total of 150 SilicoDArT markers and 108 SNP markers with high levels of differentiation between green and red skin phenotypes were identified (Bignell 2015).

A study conducted on Thai seedless sugar apple (*A. squamosa*), a spontaneous mutant, revealed that the absence of the *INNER NO OUTER (INO)* gene is responsible for seedlessness (Lora et al. 2011). The primers (LMINO), designed in this study to amplify the region or sequence immediately flanked by the *INO* locus, were further validated in the Brazilian seedless cultivar (Nassau et al. 2021). A single dominant gene controls seediness, while recessive allele combinations are responsible for seedlessness. The *INO* gene is highly conserved in seeded *A. squamosa* cultivars, and its segregation was studied previously (Rodrigues et al. 2022). A comparison of the whole-genome sequencing data of wild-type and three seedless mutants (Bs, Ts and Hs) revealed that all three seedless mutants shared the same sequence deletion at the *INO* locus. Primers (AsINODel) were subsequently designed to detect this deletion (Rodrigues et al. 2024). A combination of the two primers (LMINO 1/2 and AsINODel primers) was used to study the segregation of the locus. This codominant assay was validated using the F_1_ population obtained from a cross between Gefner (*A. × atemoya*) and Brazilian Seedless (*A. squamosa*), and the F_2_ segregating population. The observed segregation ratios were consistent with the Mendelian ratios for a single locus. Ongoing breeding programmes to develop seedless atemoya and cherimoya cultivars in Brazil and Spain actively use this marker system (Lora et al. 2018; Martin et al. 2019; Rodrigues et al. 2022, 2024).

## Genetic mapping: pinpointing QTLs for enhanced breeding

**Fig. 4:**
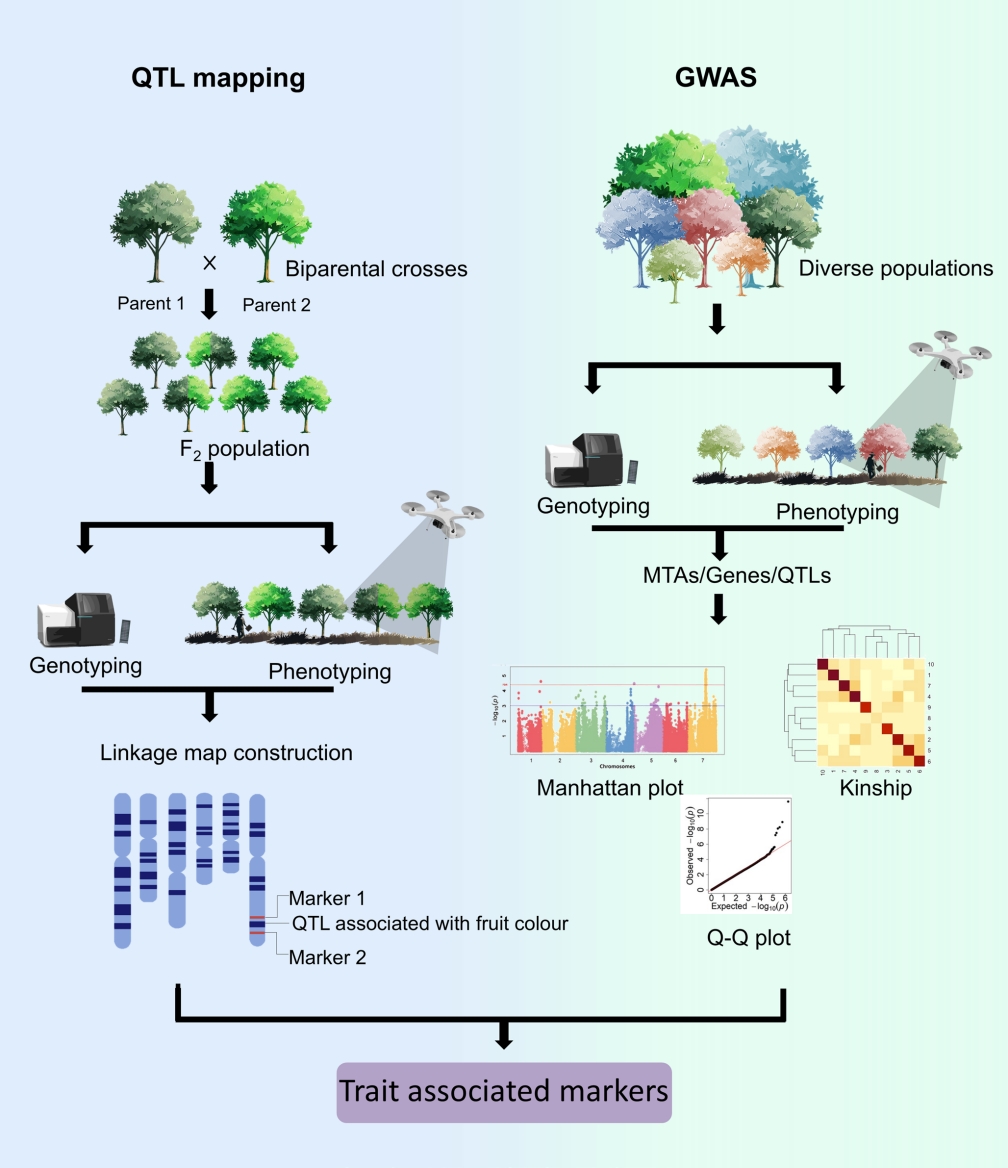
Schematic representation of QTL mapping and GWAS, outlining the key steps involved in the dissection of complex traits and identifying genetic loci underlying important agronomic traits. Created with Biorender.com

Genetic maps play an important role in crop improvement by enabling researchers to pinpoint the quantitative trait loci (QTLs) that influence important traits. These maps provide insights into the genetic architecture of complex traits, enabling the pyramiding of multiple genes and map-based cloning of genes for superior agronomic performance. Although two studies have constructed linkage maps for *A. cherimola* utilising the isozyme marker system (Lee and Ellstrand 1987; Perfectti and Pascual 1998b), the limited number of isozyme markers restricts genome coverage, thus reducing their effectiveness. However, no such studies are available for other *Annona* species. Therefore, the availability of genomic resources will aid in the discovery of many SSRs and SNPs, which can be utilised in the construction of high-density linkage maps in various *Annona* species. Such high-density maps with numerous markers will increase the mapping resolution, thus enabling precise identification of gene location, efficient gene cloning, and subsequent utilisation in *Annona* breeding programmes (Fig. 4). Moreover, these maps will be instrumental in accelerating the breeding process and enhancing efficiency by facilitating marker-assisted breeding, accelerating the selection of desirable traits based on associated genetic markers.

A crucial prerequisite for QTL mapping is the development of biparental mapping populations; these populations are typically developed using two diverse genotypes that contrast in one or more specific phenotypic traits and exhibit adequate genetic diversity to ensure a sufficient number of polymorphic markers. Equally important is the size of the mapping population: a large population increases the likelihood of recombination events, ultimately resulting in a more accurate and high-resolution genetic map. Therefore, breeding programmes should prioritise the development of biparental mapping populations and leverage existing populations for linkage map construction and QTL analysis. Integrating these efforts with genomic resources will enable the development of high-density maps for their use in QTL mapping, ultimately enhancing marker-assisted selection (MAS) and accelerating breeding efforts.

## Genome-wide association studies: high-resolution mapping for precision breeding

QTL mapping involves the development of mapping populations, a process that is both time-consuming and costly. In addition, QTL mapping has low resolution but high power (Gupta et al. 2014). An alternative mapping approach known as genome-wide association studies (GWAS) has proven effective in overcoming the limitations of pedigree-based QTL mapping. GWAS uses association mapping to correlate genotypes and phenotypes across a large number of individuals from diverse populations, including wild accessions, germplasm collections, and elite cultivars. By leveraging historical recombination events accumulated over generations, GWAS enables higher-resolution mapping, greater marker density, and more detailed dissection of the genetic basis of complex traits. With the reduction in sequencing cost, the availability of reference genomes, and different methodologies for reduced representation sequencing, a dense array of genome-wide genetic markers can now be identified and used in genomics-assisted breeding programs (Fig. 4). Efforts to utilise this powerful tool for *Annona* crop improvement are underway. Genotyping-by-sequencing (GBS) technologies are being utilised to identify trait-associated markers in the Spanish cherimoya germplasm collection (Hormaza et al. 2020). Thus, characterising existing germplasm collections maintained by various institutions as a part of breeding programs or conservation efforts is vital, as these resources offer valuable tools for genetic improvement. This approach will help dissect valuable genetic variations, thus providing new genetic variability to be exploited by breeders. These approaches will hasten the genetic improvement of Annonas.

## Decoding *Annona*: the rise of genomic resources

The number of plant genomes sequenced has steadily increased in recent years due to the decline in sequencing costs and advances in sequencing technologies. Improvements in speed, accuracy, and bioinformatics tools for assembling genomes over the past decade have made sequencing more accessible.

The *A. muricata* (soursop) genome was the first to be sequenced in the genus *Annona* (Strijk et al. 2021). This chromosomal-level genome assembly is 639.6 Mb in size, with a scaffold N50 length of 93.2 Mb. The genome contains a repeat content of 54.87% and a total of 23,375 protein-coding genes distributed across seven chromosomes. An interesting feature of the soursop genome is its low heterozygosity rate of 0.06%. Furthermore, the rich gene content includes a diverse set of genes related to plant defence and disease resistance, suggesting the potential for breeding soursop varieties with increased resistance to pathogens (Strijk et al. 2021).

The genome of the tetraploid *A. glabara* (pond apple) was sequenced as a part of a comprehensive study on the evolution of coastal mangrove forests (He et al. 2022). A combination of high-throughput chromosome conformation capture (Hi-C) and Pacific Biosciences (PacBio) high-fidelity (HiFi) single-molecule real-time (SMRT) sequencing data produced a chromosomal-scale assembly of 1027.31 Mb. Genome annotation identified 40,456 protein-coding genes, with repetitive sequences comprising 58.39% of the genome (He et al. 2022).

The genome for *A. montana* (mountain soursop) was sequenced in 2023 (Tang et al. 2023). The 973.54 Mb assembly has a contig N50 length of 7.89 Mb, anchored to seven pseudomolecules. Genome annotation revealed a total of 26,399 protein-coding genes, with repetitive sequences accounting for 61.58% of the genome. The MADS-box gene family was analysed to understand the mechanism governing flower development, flowering time and fruit development. This study identified 46 MADS-box genes classified into types I and II. RNA-Seq analysis revealed 6,537 differentially expressed genes (DEGs) associated with fruit development, including MADS-box genes and genes involved in sugar metabolism and fruit softening. An analysis of the lipoxygenase pathway revealed that volatile alcohols are the major aroma components in mountain soursop (Tang et al. 2023).

In the same year, the first *A. cherimoya* (cherimoya) genome was developed for the Spanish cultivar ‘Fino de Jete’ (Talavera et al. 2023). The assembly, totalling 1.13 Gb with a scaffold N50 length of 170.86 Mb, captures 92% of the estimated genome size and is assembled into seven pseudomolecules. A relatively high level of heterozygosity observed (1.05%) indicates that there has been limited human selection, which may be advantageous for breeding. Repetitive sequences account for 65.35% of the genome, and 41,413 protein-coding genes were identified, both values exceeding those of *A. muricata*. However, both genomes contain a higher number of genes involved in plant defence. Furthermore, no recent whole-genome duplication event has been identified, consistent with other magnoliid species (Talavera et al. 2023).

Recently, the genome of the American cherimoya cultivar (*A. cherimola* cv. Booth) was sequenced (Li et al. 2024). The Booth genome assembly, comprising 794 Mb and exhibiting a scaffold N50 of 97.59 Mb, was effectively anchored to seven pseudomolecules. A notable characteristic of this assembly is its exceptionally high proportion of repetitive elements, which constitute 68.23% of the genome, representing the highest percentage reported among all currently sequenced *Annona* genomes. A comparative genomic analysis with the Spanish cherimoya (cv. Fino de Jete) genome indicated that the Booth genome is significantly smaller by 343 Mb. The size discrepancy is largely due to variations in repetitive DNA content: the Fino de Jete genome encompasses 743.28 Mb of repeats, whereas the Booth genome includes 539.92 Mb. Thus, the Fino de Jete genome has 203.36 Mb more repetitive sequences than the Booth genome, representing 58.9% of the noted inter-genomic size variance. Despite these differences, both genomes exhibit significant nucleotide sequence identity and chromosomal alignment, except for two distinct structural variations in chr4 and chr6 of the Booth genome. A total of 45,272 protein-coding genes were identified, and gene content analysis revealed a possible loss of genes in Fino de Jete, as most of the genes present in Fino de Jete are also found in Booth, but the reverse is not true. Similar to Fino de Jete, no evidence of a recent whole-genome duplication event was found. The high divergence time (1.93 million years ago) identified between Booth and Fino de Jete suggests different origins and domestication histories. This study also identified a potential gene cluster involved in the biosynthesis of tetrahydrofuran (THF) acetogenin, a characteristic compound of the Annonaceae family with antitumour properties. Additionally, the authors attempted to develop a haplotype-phased genome assembly. The size of the obtained haplotypes was very close to the size of the draft genome (749.5 Mb and 700.5 Mb); however, the quality of the assembly was poor (Li et al. 2024).

The genome of a wild-type *A. squamosa* was sequenced recently. A combination of PacBio and Illumina reads was used to develop a genome assembly of 707.7 Mb with an average contig length of 201 kb (Rodrigues et al. 2024). Additionally, a scaffold-level genome of *A. squamosa* was sequenced as part of an initiative to sequence multiple plant species from the Yucatan Peninsula. A total of 24.8 Gb of sequencing reads generated via the Illumina HiSeq X Ten platform was used to develop a final assembly of 587.8 Mb comprising 64,832 scaffolds, with a scaffold N50 value of 58 Mb (Peña-Ramírez et al. 2024). More recently, a chromosomal-level genome of *A. squamosa* was published. The 737 Mb genome is characterised by low heterozygosity (0.18%) compared with other *Annona* species, with only *A. muricata* displaying a lower value of 0.06%. Repetitive sequences account for 64.82% of the genome, and 40,435 protein-coding genes were identified, with 88% functionally annotated. Gene family analysis revealed the expansion of gene families involved in photosynthesis, oxidative phosphorylation, and thermogenesis. Comparative evolutionary analysis revealed adaptive evolution in amino sugar, nucleotide sugar, and sucrose metabolism genes, suggesting increased hexose sugar accumulation, which contributes to the unique sweetness of *A. squamosa*. Additionally, adaptive evolution in genes involved in amino sugar, nucleotide sugar, and sucrose metabolism pathways was revealed by comparative evolutionary analysis. Further analysis of the *SWEET* gene family revealed that *SWEET* transporters involved in sucrose and fructose transport exhibit higher expression levels compared to those responsible for sucrose transport. This indicates that adaptive evolution may have enhanced selective pressure favouring fructose and glucose transport over sucrose transport (Bisht et al. 2024).

**Fig. 5:**
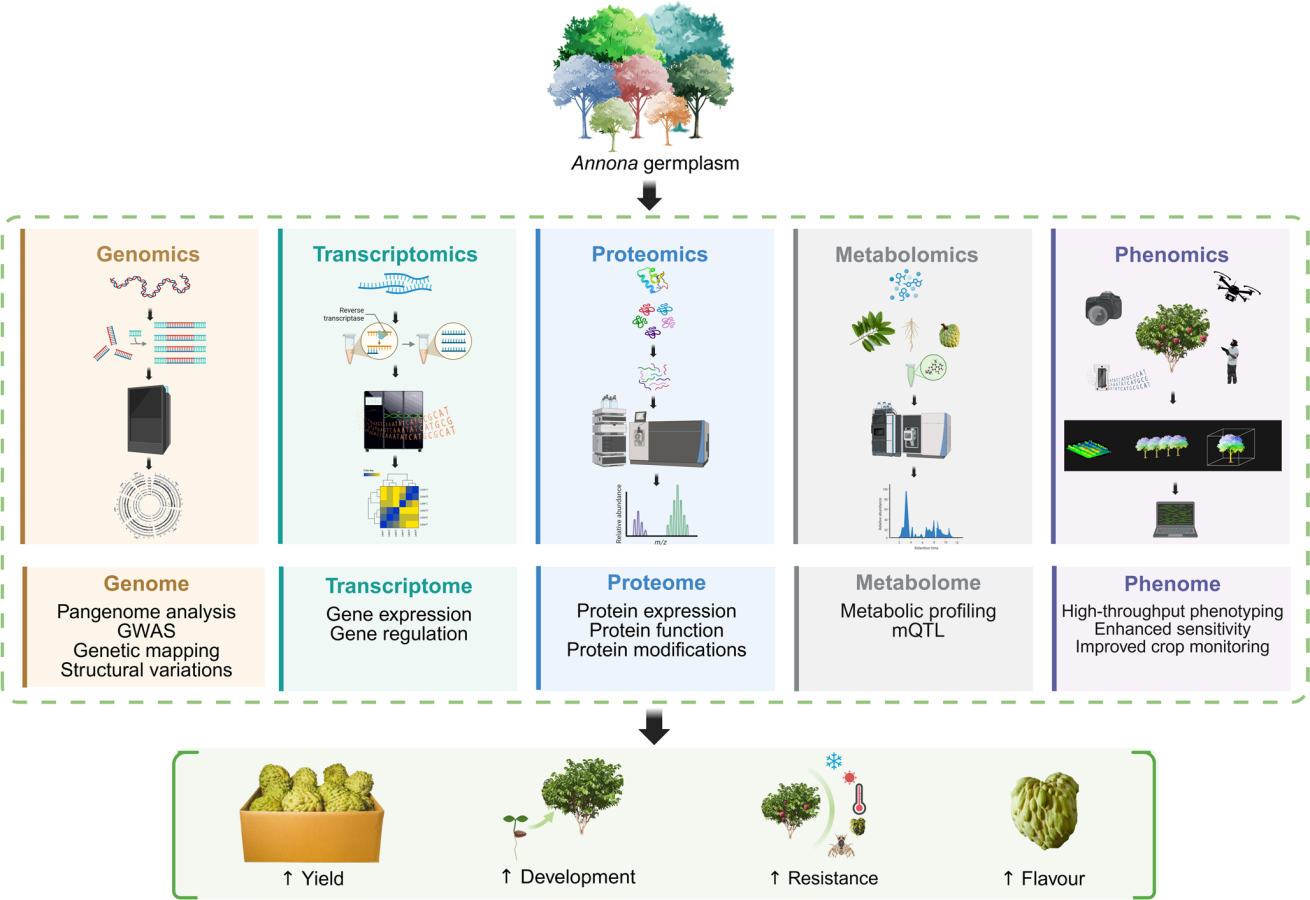
Schematic representation of the modern crop breeding paradigm, leveraging the power of multiomics technologies. Beginning with the collection of diverse germplasms, which constitute a vast reservoir of genetic variation. Subsequently, multiomics technologies have been employed to analyse the molecular makeup of these plants comprehensively. The resulting complex data sets are then integrated and analysed to identify key genetic and molecular factors associated with traits of interest. These are then translated into powerful molecular markers that enable efficient marker-assisted selection, accelerating the development of elite crop varieties. Created with Biorender.com

An overview of *Annona* genome assembly and annotation statistics published to date is presented in Table 2. The observed variation in genome size indicates a more complex evolutionary history within the genus. However, for underutilised crops such as *Annona*, there is a clear paucity of genomic resources. Among the available genomic resources, only raw sequencing reads of *A. muricata* are publicly accessible, while the genome assembly and annotation data are unavailable. Additionally, no haplotype-resolved assemblies are available to date, and no whole-genome assemblies have been reported for *A.* × *atemoya*,* A. reticulata*, *A. diversifolia*, or *A. mucosa*. Addressing these gaps will be crucial for advancing *Annona* genomics (Fig. 5).

**Table 2:** Statistics of available *Annona* genome assemblies

Assembly	*A. muricata*	*A. cherimola* ‘Fino de Jete’	*A. cherimola* ‘Booth’	*A. montana*	*A. glabra*	*A. squamosa*	*A. squamosa*	*A. squamosa*
Reference	Strijk et al. (2021)	Talavera et al. (2023)	Li et al. (2024)	Tang et al. (2023)	He et al. (2022)	Rodrigues et al. (2024)	Peña-Ramírez et al. (2024)	Bisht et al. (2024)
Total length (Mb)	656.81	1,137.39	794.02	937.54	1,027.3	707.7	587.8	737.4
Number of scaffolds	755	2052	1377	562	-	-	64,832	58
Number of chromosomes	7	7	7	7	14	7	7	7
Longest scaffold (Mb)	122.62	212.25	128.57	-	-	-	-	137.68
Scaffold N50 (Mb)	93.20	170.85	97.59	-	75.19	-	58	93.21
GC content %	40.07	34.69	35.25			-	34	34.32
BUSCOs %	-	95.6	98.43	90.80	97.65	-	-	94.1
Heterozygosity %	0.06	1.05	1.14	~ 2.4	-	-	-	0.18
**Annotation**								
Repeat sequences %	54.87	64.96	68.23	61.58	58.39	-	-	64.82
Number of protein-coding genes	23,375 (≥ 100 aa)	41,413 (≥ 50 aa)	45,272 (≥ 30 aa) *	26,399 (≥ 50 aa)	40,456**	-	-	40,435 (≥ 50 aa)
Number of genes with annotation	22,769	-	32,377	25,933	-	-	-	-
BUSCO%	92.14	90.9	92.0	96.59	97.40	-	-	88.3

*43,926 (≥ 50 aa), 34,890 (≥ 100 aa)

** amino acid cut-off not available

To enhance genomic resources for *Annona* species, future studies should prioritise improving genome assembly quality using advanced long-read sequencing technologies (e.g., PacBio HiFi, Oxford Nanopore) and chromosome conformation capture techniques (e.g., Hi-C). Gapless, telomere-to-telomere assemblies will provide deeper insights into the evolution, domestication, and adaptation of *Annona* species (Garg et al. 2024).

## Pangenome: a comprehensive catalogue of genetic diversity

Numerous plant species possess complex genomes characterised by large sizes, high heterozygosity, and polyploidy, which present considerable challenges for genome assembly. Furthermore, the open-pollinated nature of many plants results in considerable variations in their phenotypes and influences various traits (Sun et al. 2020). Sequencing multiple lines will enable the identification of genetic variants, beneficial for breeding programs. However, while a single reference genome is valuable for marker discovery, it often falls short of capturing the vast genetic diversity present in these plant species (Varshney et al. 2021). This limitation arises from the prevalence of intraspecific variation, particularly structural variants, which can significantly impact phenotypic traits (Wang T et al. 2023).

The construction of a pangenome, which involves the sequencing and integration of multiple genomes, offers a more comprehensive approach to mitigate this issue. A pangenome consists of a core genome, encompassing the genes present in all accessions, and a dispensable genome, which contains genes shared by a subset of the accessions. This approach facilitates the identification of genome-wide variations associated with different traits and provides deeper insights into genetic diversity and the molecular basis of traits. Furthermore, comparative analysis of core and dispensable genomes can illuminate the impact of domestication, identify genes absent from reference genomes and further resolve taxonomic and domestication challenges (Tan et al. 2024). For example, the papaya pangenome study revealed 34 Mb of chromosome region, encompassing 1185 genes, which displayed strong domestication selection signals. Among these, two genes were identified as contributing to increased papaya yield in domesticated varieties. Comparisons of gene frequencies showed that genes related to stress response and terpene synthesis activity were reduced in frequency or lost in cultivated populations, likely influencing traits like fruit flavour. Conversely, genes involved in fruit quality and ripening were positively selected (Yang et al. 2025). In bacteria, where horizontal gene transfer is prevalent, species differentiation can be very difficult. Pangenome analysis has been employed to clarify taxonomic ambiguities in genera such as *Bradyrhizobium*. Examining the pangenome of various *Bradyrhizobium* species allows for the identification of genes associated with nodulation and nitrogen fixation. This analysis reveals how the presence, absence, or variation of these genes correlates with different species or ecotypes, offering insights into their evolutionary origins and genetic diversity (Terra et al. 2025).

This approach is particularly valuable for genetically diverse crops, where a single reference genome may not adequately represent the population’s genetic complexity, thus aiding in the capture of the full spectrum of genetic variation for effective breeding programs. The development of pangenomes in fruit species such as *Malus* (apple) (Sun et al. 2020; Wang T et al. 2023), *Musa* (banana) (Rijzaani et al. 2022), *Prunus* (apricot) (Tan et al. 2024), *Vitis* (grape) (Guo et al. 2024), and *Citrus* (Huang et al. 2023) has facilitated the discovery of numerous candidate genes and alleles associated with key agronomic traits that were not previously accounted for in the original reference genomes. Furthermore, the recent pangenomes of *Fragaria* spp. (strawberry) (Qiao et al. 2021), *Vaccinium* genus (blueberry and cranberry) (Yocca et al. 2023) revealed that dispensable genes accounted for ~ 50% of the genome related to adaptive environmental responses, which would have been missed in a single-reference approach. Despite its great potential, efforts to develop pangenome research remain a future goal in *Annona*. Our research team at Murdoch University, in collaboration with The University of Queensland and Queensland Department of Primary Industries, is pioneering efforts to develop the first *Annona* pangenome as a part of the Hort Innovation (Australia)-funded “Advanced Genomic Platform for Australian Horticulture Project”. The availability of a pangenome will enable the capture of extensive genetic diversity in *Annona* species and facilitate the development of pangenomic markers. These markers will facilitate the identification and transfer of desirable alleles within *Annona* species, reducing linkage drag and shortening breeding cycles.

## Beyond genomics: exploring multiomics innovations for greater trait understanding

### Deciphering crop biology: the power of transcriptomics in trait dissection

The transcriptome encompasses the complete set of transcripts generated by a cell. This includes every type of RNA transcript, including messenger RNAs (mRNAs), microRNAs (miRNAs), and various long noncoding RNAs (lncRNAs). Transcriptomics involves the comprehensive analysis of the transcriptome. This includes identifying molecules, examining their transcription and expression levels, functions, subcellular locations, trafficking pathways, and degradation processes (Wang et al. 2009). Transcriptome analysis has significantly advanced our understanding of genes and pathways involved in different biological processes. Advances in NGS technologies and bioinformatics tools have made RNA-seq a widely utilised method in transcriptomics. Since *de novo* assembly does not require a reference sequence, the application of RNA-seq has broadened to minor/orphan crop species lacking genome sequence data and expressed sequence tags (ESTs) (Shiratake and Suzuki 2016). These findings will facilitate the development of comprehensive transcriptome profiles, aid in the identification of differentially expressed genes (DEGs), and support the development of molecular markers.

The first *Annona* transcriptome was sequenced for *A. muricata* as part of the 1 K Transcriptome Project, aimed to establish a comprehensive catalogue of plant genetic variation (Matasci et al. 2014). From 2.80 Gb of sequencing data, 40,486 transcripts were assembled. Transcriptome data were also generated as a part of several genome sequencing projects from various plant tissues for genome annotation purposes (Strijk et al. 2021; Talavera et al. 2023; Tang et al. 2023).

Transcriptomic studies have been employed to elucidate the regulation of gene expression throughout flower development, fruit development, ripening, and postharvest storage in various *Annona* species. To understand the molecular mechanisms underlying the difference in seed number, the transcriptome profiles of two *A. squamosa* genotypes, Sitaphal (high seed density) and NMK-1 (low seed density) were analysed at early fruit development stages (0, 4, 8, and 12 days after pollination) (Gupta et al. 2015). Approximately 1.9 million reads were obtained by pyrosequencing and were *de novo* assembled into 14,921 (Sitaphal) and 14,178 (NMK-1) unigenes. Functional annotation indicated that numerous unigenes associated with pathways related to primary and secondary metabolism, seed formation and fruit development were highly expressed in cv. Sitaphal, characterised by dense seed production compared to NMK-1. Furthermore, qRT‒PCR analysis revealed reduced expression of key orthologous genes, including *Clavata-3*, *Abnormal Suspensor-2*, *Embryo Defective-1144*, *Embryo Defective-2742*, and *Ovule Abortion-9* (Gupta et al. 2015). These genes play critical roles in embryogenesis, ovule development, and seed viability, highlighting their potential impact on seed number regulation in *A. squamosa* (Gupta et al. 2015).

Flower development is a crucial phase in the life cycle of higher plants, especially fruit trees. This complex process is affected by numerous genetic and environmental factors. A comprehensive understanding of the molecular mechanisms governing flower development is essential for increasing breeding efficiency. To investigate these mechanisms in *A. squamosa*, gene expression patterns were examined through transcriptome analysis of flowers at four distinct developmental stages: inflorescence meristem, flower bud, partially opened flower, and fully opened flower (Liu et al. 2016a). Using Illumina sequencing, over 107 million reads were generated and assembled into 71,948 unigenes. Among these, 42,831 unigenes were consistently expressed across all four stages, while 83.43% of the unigenes were shared in at least three stages. Functional annotation of the unigenes revealed that key metabolic pathways, such as starch and sucrose metabolism, hormone signalling, and circadian rhythms, play crucial roles in flower development (Liu et al. 2016a). Additionally, the study identified 5,903 genes encoding transcription factors (TFs), essential regulators of gene expression, and their expression patterns during flower development. The expression of the *bHLH*,* MYB*, and *bZIP* TF families increased after early flowering, while *NAC and C2H2* showed heightened expression at later stages. Conversely, *WRKY*,* ERF*, and *FAR* TF families exhibited a decline throughout flower development. Furthermore, an analysis of endogenous hormone dynamics revealed a decline in indole-3-acetic acid (IAA), gibberellins (GA) and zeatin ribosides (ZRs) as flower development progressed. However, the level of abscisic acid (ABA) increased significantly during this process (Liu et al. 2016a). These findings provide valuable insights into the molecular regulation of flower development in *A. squamosa*, offering potential targets for breeding programs aimed at optimising flowering and fruit set.

Floral malformation is prevalent in *Annona* species and can significantly affect fruit yield and quality. This condition, marked by atypical development of floral organs, can be linked to multiple factors, such as environmental stresses, genetic predispositions, or hormonal imbalances. A comparative transcriptomic analysis of normal and malformed flowers (unopened) in *A. squamosa* was carried out to identify and analyse potential genes associated with floral organ development (Liu et al. 2017). A total of 70,189,896 reads were generated and assembled into 55,097 unigenes. A total of 12,664 DEGs were identified, including 701 TF genes associated with flower development. Further analysis revealed that numerous DEGs were associated with flower development, including genes that play crucial roles in hormone signalling, the regulation of transcription factors, and the control of the cell cycle. The majority of the hormone-related DEGs were downregulated in malformed flowers. The concentrations of various hormones, such as auxin, cytokinin, and gibberellin, are lower in malformed flowers than in their normal counterparts (Liu et al. 2017). This evidence indicates that disruptions in hormonal signalling pathways could contribute to the phenomenon of floral malformation.

*Annona* species are known for their exquisite fruits that are characterised by high sugar content, which varies among different species and genotypes (Brandão and Santos 2016). Thus, understanding sugar transport, metabolism, and accumulation is vital for improving fruit quality. The full-length transcriptomes of two atemoya (*A. × atemoya*) cultivars, African Pride and Gefner, were sequenced to identify the molecular mechanisms underlying sugar accumulation (Ren et al. 2020). Fruits in more than five growth and ripening stages [30 days after pollination (DAP), 100 DAP, 130 DAP, 6 days after harvest (DAH), and 8 DAH] were selected. PacBio’s Iso-seq technology was used to generate a total of 48,209 high-quality, full-length transcripts identified from the 783,647 circular consensus sequences, comprising 1,838 transcription factors and 1,768 long noncoding RNAs. They subsequently analysed the expression patterns of genes associated with sugar transport and metabolism during the different stages of fruit development and ripening. During the initial phases, genes associated with sugar metabolism, transport, and starch synthesis were highly expressed. As the fruit matured, the expression of genes associated with sucrose production and starch degradation increased. This transition led to a substantial increase in sucrose content and a reduction in starch content. Sucrose synthase is recognised as a crucial enzyme in the regulation of sucrose import and starch accumulation during early development. Moreover, sugar transporters are essential for the translocation of sugars into fruit (Ren et al. 2020). These results suggest that starch breakdown is a major contributor to the high sugar content in the fruit.

Preharvest and postharvest fruit splitting poses a significant challenge in *Annona* species, leading to yield loss and a reduction in economic value. Preharvest fruit splitting is primarily caused by low night temperatures and/or soil moisture stress (Pinto et al. 2005; George et al. 2010). Postharvest fruit splitting appears as small cracks in the outer cuticle, usually along the radial lines and peduncle near the base, which subsequently deepen over time. This is believed to be caused by osmotic and turgor changes during ripening (Paull 1996). Three expansin genes (*AcExp1*,* AcExp2*, and *AcExp3*) were isolated and characterised from cherimoya fruit to assess their roles in fruit softening under different storage conditions (Shen et al. 2009). Gene expression was analysed at several temperatures (10 °C, 15 °C, and 20 °C) following propylene treatment (Shen et al. 2009). *AcExp2* and *AcExp3* expression increased during fruit softening, particularly under higher storage temperatures and propylene treatment. Furthermore, expression analysis of the three *AcExp* genes in vegetative tissues and young fruits revealed that all three *AcExp* mRNAs were exclusively present in soft fruits, with minimal levels of *AcExp1* and *AcExp3* mRNAs observed in young leaves. These findings confirm that the *AcExp* genes are primarily confined to fruit and involved in fruit softening (Shen et al. 2009).

Similarly, the role of xyloglucan endotransglycosylases (*XET*s) in cherimoya fruit softening was investigated by isolating and characterising three *XET* genes (*AcXET1*,* AcXET 2*, and *AcXET 3*) from cherimoya (Li et al. 2009). The expression levels of the *XET* and *EXP* genes were studied in cherimoya fruits treated with or without 1-methylcyclopropene (1-MCP), an ethylene inhibitor, and stored at 20 °C to assess the impact of 1-MCP on these genes during ripening. Compared to the control, 1-MCP treatment significantly delayed fruit softening and suppressed ethylene production. Compared to *AcXET3*, the expression of the *AcXET1* and *AcXET2* genes peaked later in the ripening process, while *AcEXP1* and *AcEXP3* peaked earlier than *AcEXP2*. Furthermore, 1-MCP treatment postponed the expression of all *XET* and *EXP* genes, reinforcing their role in ripening regulation (Li et al. 2009). A complementary study evaluated the effects of citric acid and chitosan coatings on the *XET*,* EXP* and pectin esterase (*PE*) genes during cherimoya fruit ripening (Liu et al. 2016b). The expression levels of *AcXET2*, *AcEXP1*, *AcEXP3*, and *AcPE* were greater during the early ripening stage, whereas *AcXET1* and *AcEXP2* expression levels peaked during the later stages (Liu et al. 2016b). Additionally, citric acid and chitosan treatment significantly reduced the mRNA expression of *AcXETs*,* AcEXPs*, and *AcPE*, delayed the accumulation of *AcXET1* and *AcEXP2* and accelerated the decrease in *AcXET3*, suggesting a role in inhibiting fruit softening (Liu et al. 2016b). Similar to previous studies, *EXP* genes were confirmed to be associated with the ripening process of soursop (*A. muricata*) fruit (Berumen-Varela et al. 2020). In addition to *EXP* genes, *Succ-CoA* and *ALDH* genes were upregulated during soursop ripening, indicating their functions in cellular instability, organic acid metabolism, and acetaldehyde synthesis.

A recent study to elucidate the key metabolic pathways and gene expression patterns involved in cell wall polysaccharide disassembly and postharvest fruit softening in *A. squamosa* revealed 41,545 nonredundant unigenes and 7,571 DEGs (Wang S et al. 2023). These genes were involved in various metabolic pathways related to cell wall degradation, including starch and sucrose metabolism, galactose metabolism, and pentose and glucuronate interconversions (Wang S et al. 2023). During ripening, the upregulation of cellulase (*Cx*), polygalacturonase (*PG*), pectin methylesterase (*PME*), neutral xylanase (*NEX*), β-galactosidase (*β-Gal*) and β-glucosidase (*β-Glc*) genes promoted the degradation and solubilisation of cell wall polysaccharides, resulting in fruit softening.

Transcriptome analysis of soursop cv. GUANAY-1 during different stages of fruit ripening (0, 3, and 9 days after harvest at 28 ± 2 °C and 0, 3, 6 and 9 days after harvest at 15 ± 2 °C) revealed increased expression of *PE*,* PG*,* EXP*, and pectate lyase (*PL*); these enzymes contribute to fruit softening by breaking down pectin. Storage of the fruit at 15 °C delayed the expression of pectin degradation enzymes, thus prolonging shelf life. Network analysis revealed that *PE* genes are major regulators of fruit softening (Palomino-Hermosillo et al. 2022).

A transcriptome sequencing study on atemoya cv. African pride was conducted to understand postharvest fruit cracking (Chen et al. 2019). Samples were collected from multiple fruit stages exhibiting varying degrees of cracking and ethylene treatment (recently harvested fruits, fruits with pedicel cracking, fruits with moderate pedicel cracking, fruits with severe pedicel cracking, fruits 24 h post ethephon treatment, fruits with pericarp cracking post ethephon treatment, and fruits without cracking post ethephon treatment). Using the Illumina HiSeq 2500 platform, sequencing generated 50.9 million, 57.1 million, 49.4 million, 50.0 million, 69.58 million, 47.4 million, and 38.2 million reads, respectively. Pathway and enrichment analyses of the DEGs revealed 39 DEGs associated with starch and sucrose metabolism. RT-qPCR validation identified the key genes involved in starch, pectin, and cellulose degradation. The results revealed that *AMY1*,* AMY3*,* BAM1*,* BAM3*,* BAM9*,* PUL*, and *glgX* are the principal genes involved in starch degradation. In contrast, *GAUT* and *GBSS* are involved in protopectin production, and *PE*,* PG*, and *PEL* are associated with pectin degradation. The expression of genes involved in starch degradation, such as alpha-amylase and beta-amylase, was elevated during the cracking process. The inhibition of gene expression related to protopectin production indicated a reduction in cell wall strength. Thus, the upregulation of starch degradation enzymes and the downregulation of protopectin production genes likely facilitate the weakening of cell walls, resulting in cracking (Chen et al. 2019).

In a related study, the transcriptomes of split and non-split atemoya fruits ten days after harvest were sequenced, and several DEGs associated with starch and sucrose metabolism were identified, indicating their role in fruit splitting (Li et al. 2019). Hormone signalling was also implicated, with the majority of auxin-related DEGs downregulated in split fruits, likely affecting cell wall formation. Abscisic acid (ABA), cytokinin, gibberellins (GA), and salicylic acid-related DEGs were upregulated, whereas DEGs related to jasmonic acid were downregulated, indicating complex hormonal interactions influencing fruit cracking. The upregulation of DEGs associated with cytoskeleton organisation, plant cell wall biogenesis, cell wall thickening, and α-galactosidase activity suggested a positive correlation between cell wall weakening and fruit splitting. Additionally, the expression of an *EIN3-like* gene associated with ethylene signalling was significantly altered in split fruits, indicating the possible involvement of this gene in splitting (Li et al. 2019). The results from both studies consistently highlight that fruit splitting in atemoya is driven by starch degradation, reduced cell wall integrity, and hormonal imbalances. These insights provide a foundation for postharvest management strategies and breeding programs aimed at reducing fruit cracking,

### Proteomics: exploring the protein dynamics

Proteomics offers a comprehensive understanding of the proteins translated from genes by analysing protein expression, modifications and interactions (Weckwerth et al. 2020). Identifying specific protein expressions, modifications, and interactions associated with important traits/pathways is crucial, as this knowledge can facilitate precision breeding by enabling the targeted manipulation of proteins to improve crop yield, quality, and resistance to biotic and abiotic stresses (Weckwerth et al. 2020). Further analysis of these proteomic profiles will enable the identification of protein markers associated with desirable traits, which can be utilised for crop improvement strategies.

*A. muricata* possesses numerous medicinal properties (Brisbie et al. 2017). Annomuricatins, a group of cyclic peptides, are orbitides with cytotoxic and anti-inflammatory properties. To understand the biosynthesis of annomuricatins derived from *A. muricata* seeds, a combination of *de novo* transcriptomics and tandem mass spectrometry, was used to identify genes encoding their precursors and sequence the mature peptides (Fisher et al. 2020). A total of ten annomuricatins were identified, with nine novel discoveries and one previously recognised from *A. muricata* seeds. The genes responsible for generating the annomuricatin precursors included six highly identical short genes. Among these, four genes were found to encode two cyclic peptides each, while the remaining two genes encoded a single peptide. Subsequent studies of publicly accessible transcriptome data indicated that these genes are also present in the leaves of *A. muricata* and the flowers of *A. squamosa*, suggesting that such cyclic peptides may also exist in other Annonaceae species (Fisher et al. 2020).

A protein expression study was used to understand the mechanism behind sugar apple (*A. squamosa*) flower development (Dong et al. 2018). An analysis of the proteome across four distinct flower stages revealed more than 800 proteins at each stage, with 50 demonstrating significant variations in expression. Proteins related to energy metabolism were found to be crucial, such as ATP synthase subunits and pyruvate decarboxylase, exhibiting increased expression, while proteins related to secondary metabolism, stress and defence also play pivotal roles. Additionally, two novel tetratricopeptide repeat-containing proteins with unknown functions in sugar apple flower development were identified in this study (Dong et al. 2018). These findings reveal that flower development involves a complex interplay of various proteins.

Expanding proteomic research across a broader range of *Annona* species is imperative to broaden our understanding of the different proteins involved in controlling key traits and pathways, such as those involved in fruit quality and development, stress tolerance and adaptation. A comprehensive proteomic study, integrated with other omics data, will provide a deeper understanding into the molecular intricacies and diversity within the genus, ultimately improving knowledge and aiding the development of improved cultivars (Fig. 5).

### Metabolomics: fingerprinting the chemical cascades

Metabolomics, the study of small-molecule metabolites, enables a deeper understanding of the complex genotype-phenotype relationships in plants. By analysing metabolomic variations across different plant samples, researchers can identify key metabolic pathways and metabolites associated with different traits (Weckwerth et al. 2020). This approach facilitates the identification of metabolomic biomarkers that can be used for precision breeding. Additionally, researchers can understand the complex interactions among genes, gene expression, and metabolic processes through the integration of metabolomic data with genomic and transcriptomic data. This integration facilitates the identification of QTLs that regulate metabolite accumulation.

Metabolite Quantitative Trait Locus (mQTL) refers to the genomic region associated with the regulation and synthesis of primary and secondary metabolites (Pott et al. 2020). Since metabolites influence many important traits, including fruit quality and aroma, by identifying the mQTLs for these metabolites, it is possible to delineate the particular genomic region responsible for the control of these traits. This can be further utilised to develop markers applicable in breeding programs, thus facilitating the selection of plants with preferred characteristics (Pott et al. 2020; Bilbrey et al. 2021). The identified mQTL regions can also be utilised to pinpoint candidate genes associated with the biosynthesis, transport, and regulation of these metabolites.

The genus *Annona* is well known for its diverse phytochemical profile, with numerous studies characterising these compounds in different plant tissues, as comprehensively reviewed in multiple studies (Coria-Téllez et al. 2018; de Moraes et al. 2020; Kazman et al. 2020; Oliveira et al. 2021). In one such study, the phenolic profiling of cherimoya (pulp, peel and seed) revealed a rich diversity of phenolic compounds, including flavan-3-ols (Catechin, Epicatechin, and Procyanidins) and organic acids. The peel was identified as the most abundant source of phenolic compounds, whereas the seeds were identified as a rich source of organic acids (García-Salas et al. 2015). Similarly, a recent study catalogued 68 metabolites in soursop fruit during ripening, including phenolic compounds, flavonoids, organic acids, and polyphenols. The abundance and type of metabolite profile were directly related to the ripening stage (Ochoa-Jiménez et al. 2024). To gain deeper insights into the molecular mechanisms underlying soursop metabolism during ripening, a comprehensive study of previously published transcriptomic (Palomino-Hermosillo et al. 2022) and metabolomic (Ochoa-Jiménez et al. 2024) data were integrated (Palomino-Hermosillo et al. 2024). The primary pathways involved in this process are starch and sucrose metabolism, which influence fruit ripening. Numerous phenolic compounds (Kaempferol 3-O-galactoside, Procyanidin C1, Procyanidin trimer C1, and m-Coumaric) and genes (*UBL5_ARATH*, *FTSH8_ORYSJ*, *ZIP4_ARATH*, *TRL31_ORYSJ*, *YODA_ARATH*, *MGL_ARATH*, *PRS6_SOLLC*, *C7A13ARATH*, *C84A1_ARATH*, and *MET2_ORYSJ*) exhibited correlations and differential expression during this process (Palomino-Hermosillo et al. 2024). A more recent study investigated the prospects of integrating metabolomics and machine learning to identify biomarkers to assess fruit quality changes during postharvest storage (Zhang et al. 2025). Twenty potential biomarkers related to reactive oxygen species regulation, energy metabolism, membrane integrity, aroma/flavour-related, and general metabolism have been identified (Zhang et al. 2025). These biomarkers could be utilised to develop non-destructive maturity assays, shelf life predictions, fruit quality grading and sorting strategies, optimise storage and handling strategies, and provide valuable insights into low-temperature fruit preservation. Further cataloguing the metabolome of various plant parts during important stages and correlating the results with other omics technologies will help us understand these biological processes in detail (Fig. 5). Such efforts will improve our knowledge regarding important biological processes and aid in making informed breeding decisions.

### Genetic engineering: precision breeding for climate-smart cultivars

Climate change-driven pest and disease incidences and shifts in climatic conditions pose a high threat to the fruit industry. Although effective, traditional breeding methodologies are often too slow to address these emerging threats. In recent years, plant genetic engineering has emerged as a powerful tool for crop improvement, enabling the rapid development of cultivars with enhanced yield, stress tolerance, and improved nutritional profiles (Zhang and Zhu 2024). Since the development of the “FlavrSavr” tomato, the first genetically modified crop (Kramer and Redenbaugh 1994), to the first genetically modified banana (Turrell 2024), revolutionary progress in plant biotechnology has also been fuelled by the discovery of trait-associated alleles and genes. In the past decade, the potential of genetically modified and gene-edited crops has been demonstrated in various species, increasing yield and improving other vital traits, such as biotic and abiotic stress tolerance and the nutritional profile of the crop (Zhang and Zhu 2024). Notably, the SunUp and Rainbow papaya cultivars, which exhibit resistance to papaya ringspot virus, are pivotal in reviving the declining papaya industry (Subejano et al. 2024).

Thus, genetic engineering holds high prospects for fruit crop improvement. However, there are challenges to be addressed. Identifying trait-associated genes and developing efficient transformation methods are major bottlenecks (Sharma et al. 2023). Advancements in sequencing technologies have paved the way for the accelerated discovery of gene‒trait relationships. However, many fruit trees lack comprehensive genomic resources, hindering the precise identification of target genes (Savadi et al. 2021). Studies on genetic transformation in *Annona* species are limited and have primarily focused on controlled fruit ripening and seedlessness. Early studies explored the possibility of *Agrobacterium*-mediated transformation of atemoya and cherimoya, with recent success in developing a protocol for cherimoya hypocotyl explants (Hormaza et al. 2020). The availability of multiomics resources can expedite the identification of potential target genes for genome editing. By integrating multiomics data, researchers can gain valuable insights into gene function and regulation, enabling precise selection of the target genes controlling a specific trait of interest (Fig. 5). This approach can significantly accelerate the development of new climate-resilient cultivars tailored to withstand environmental challenges and improve fruit production.

### Genomics-assisted breeding: breeding cultivars better and faster

The integration of multiomics resources is transforming genomics-assisted breeding, enabling the adoption of genomic selection (GS) and MAS. While MAS focuses on the transfer of one or two major genes for specific traits, GS leverages genome-wide markers to predict the genetic value of an individual for complex traits. Furthermore, a deeper understanding of the underlying biological processes for a trait made available from multiomics data will help enhance prediction accuracy. The sequencing of diverse germplasm and the collection of comprehensive phenotypic data can identify haplotypes associated with key traits. By developing haplotype-defining DNA markers, breeders can develop tailored cultivars with superior performance while minimising linkage drag (Varshney et al. 2021). The application of these methodologies has the potential to revolutionise *Annona* breeding, hastening the breeding cycle and enabling the development of superior varieties with enhanced traits (Fig. 5). To fully capitalise on these innovations, the priority must be identifying robust genes and QTLs associated with important agronomic traits in *Annona*. This requires using a variety of genome mapping approaches and integrating the findings into MAS and GS pipelines. By doing so, breeders can develop next-generation *Annona* with enhanced productivity, resilience, and fruit quality, ensuring sustainable production in the face of evolving agricultural challenges.

### From genetic complexity to genomic solutions: accelerating breeding in the face of challenges

**Fig. 6:**
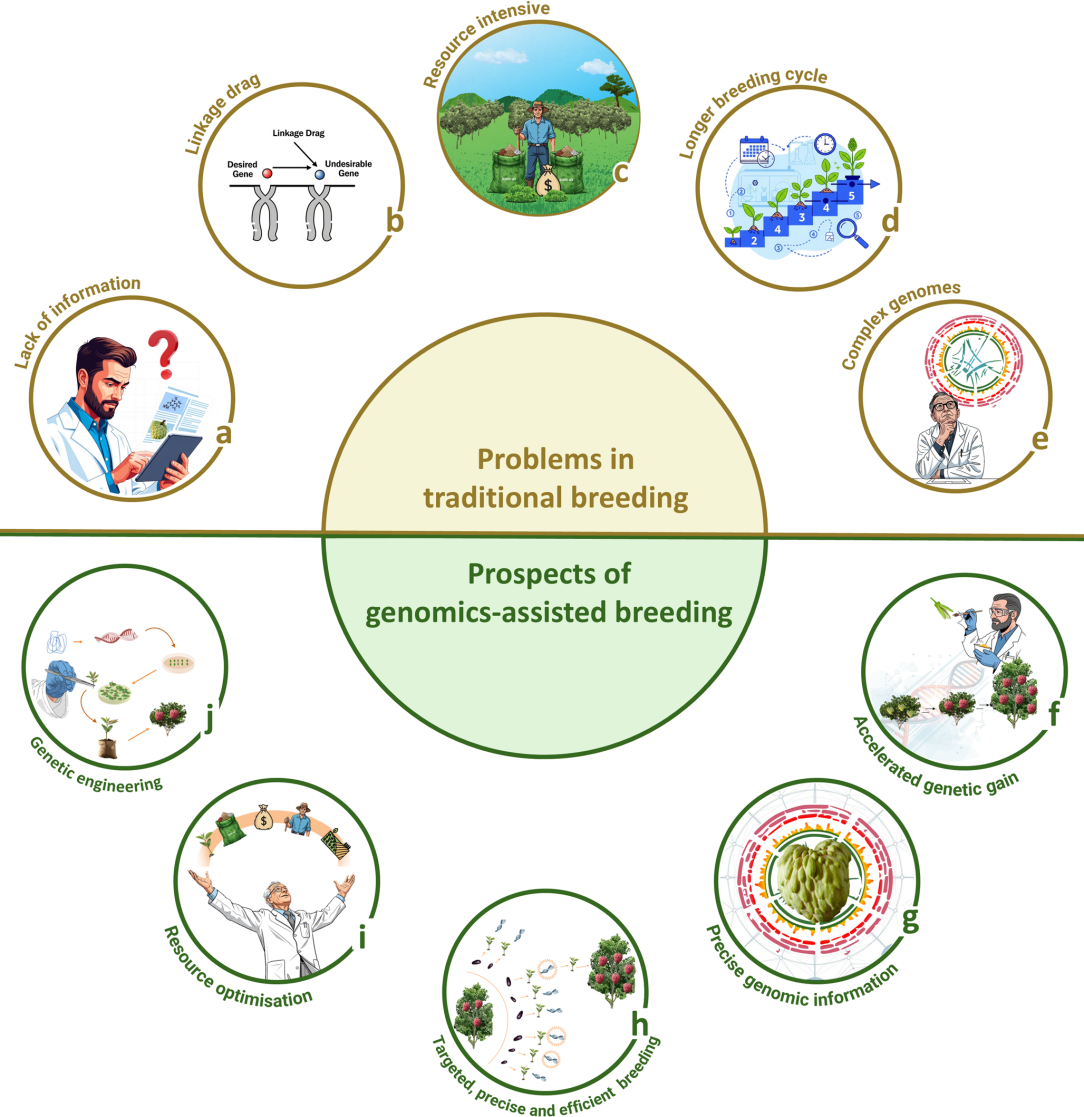
Challenges in traditional breeding and prospects of genomics-assisted breeding: The upper semi-circle illustrates the limitations of traditional breeding, including (**a**) lack of information, (**b**) linkage drag, (**c**) resource intensive processes, (**d**) longer breeding times, and (**e**) complex genomes. The lower semi-circle highlights the advantages of genomics-assisted breeding, such as (**f**) accelerated genetic gain, (**g**) precise genomic information, (**h**) targeted, precise, and efficient breeding, (**i**) resource optimisation, and (**j**) genetic engineering

*Annona* breeding and cultivation face significant challenges, including complex genetic structures, long reproductive cycles and limited genetic resources (Fig. 6). The high heterozygosity and polyploidy of many *Annona* species complicate the application of traditional breeding efforts, making genetic improvement slow and resource intensive. Moreover, breeding programmes are limited to a few countries due to challenges associated with maintaining and evaluating large populations, including high costs, land requirements and labour-intensive management. As a result, conventional breeding strategies struggle to keep pace with the growing global demand for *Annona* fruits. One of the major bottlenecks in *Annona* improvement is the lack of genomic resources, particularly high-quality reference genomes and marker-assisted selection techniques, which impede rapid advancements in cultivar development. Additionally, environmental constraints such as climate variability, soil sensitivity and susceptibility to abiotic and biotic factors, pose significant challenges and contribute to yield instability (Pinto et al. 2005; Lora et al. 2018). The limited genetic diversity in cultivated *Annona* species, combined with inadequate germplasm conservation efforts, hinder the incorporation of novel traits into breeding programs. A survey conducted by the National Network of Annonaceae (REMA) of the National Plant Genetic Resources (SINAREFI) in Mexico revealed that conservation efforts remain inconsistent, with germplasm largely limited to domestic gardens. This presents a hurdle, particularly for species that are not extensively cultivated. In addition, the recalcitrant nature of *Annona* seeds makes their long-term conservation challenging (Agustín and Ledesma 2014). This situation applies to all the countries where *Annona* species are cultivated. As breeding programmes prioritise some species, conservation efforts for other species are often overlooked. As a result, most of the *Annona* species remain underutilised, are vulnerable, and are at risk of becoming critically endangered (IUCN 2024).

Recent advancements in genomics present promising solutions to these challenges (Fig. 6). Notably, reference genomes are available for key *Annona* species, including soursop (Strijk et al. 2021), cherimoya (Talavera et al. 2023; Li et al. 2024), sugar apple (Bisht et al. 2024; Peña-Ramírez et al. 2024; Rodrigues et al. 2024), pond apple (He et al. 2022), and mountain soursop (Tang et al. 2023). The sequencing of different *Annona* species will unlock new opportunities for genomics-assisted breeding. Genome-wide association studies (GWAS) and QTL mapping will increase the identification of essential genetic loci linked to important traits. A greater understanding of the different species and their genetic makeup will also enable breeders to exploit interspecific variation effectively through genomic-assisted breeding technologies and help streamline conservation efforts. Moreover, elucidating the molecular mechanisms underlying different traits, such as fruit ripening, shelf life, and fruit cracking, as well as characterising and sequencing diverse germplasms, can facilitate the identification of desirable alleles. Ultimately, these advances will drive the development of improved cultivars and ensure faster, more precise and cost-effective breeding practices. Additionally, genetic engineering offers precise methods for altering specific genes, thereby improving fruit flavour and augmenting resistance to environmental stressors. These innovations will help *Annona* breeding keep pace with other fruit crops, ensuring the development of resilient cultivars suited for changing climates and expanding market demands.

## Conclusion

The primary focus of *Annona* cultivation currently revolves around cherimoya, atemoya, sugar apple, and soursop, which are widely recognised for their commercial value as the most popular fruit crops among Annonas. However, additional *Annona* species hold untapped potential and have the possibility to become new crops in specific regions across the globe. Despite their diversity, many species remain underutilised and are poorly understood. This highlights the need for further research to bridge this gap and expand their cultivation and breeding potential. The availability of genomic resources for *Annona* species and the integration of genomic tools such as GWAS, MAS, and GS can potentially transform the efforts to enhance efficiency and precision in developing superior cultivars. Moreover, effective germplasm conservation and thorough characterisation are essential for ensuring the long-term success of *Annona* breeding. A complementary strategy that utilises genomic innovations while maintaining genetic diversity, bolstered by efficient data management and dissemination, is essential for unlocking the complete potential of *Annona* species and securing their future as valuable fruit crops.

### Future prospects

Traditional breeding methods have contributed to genetic improvement efforts by developing novel cultivars and selections. However, to keep pace with the growing market demand and changing environmental conditions, it is essential to concentrate on several key areas that necessitate coordinated efforts to enhance *Annona* improvement. Firstly, producing high-quality genomes and annotations for various *Annona* species is essential, as the quality of these data directly influences their applicability. Secondly, there is a need for more functional studies to elucidate the role of genes and regulatory networks within *Annona*, as this can offer profound insights for focused breeding. Thirdly, systematic efforts to characterise current germplasm collections for important traits and establish a standardised approach for phenotyping data collection to enhance comparability and data utilisation. Furthermore, efforts to actively conserve germplasm collections by utilising current resources and knowledge, as well as developing and implementing newer, more effective protocols for short, medium and long-term preservation of germplasm. In addition to data collection, the implementation of efficient data management and sharing strategies will be essential to optimise the utilisation of these valuable resources. It is essential to tackle the issue of obtaining sustainable funding for research programmes and the long-term maintenance and preservation of germplasm collections. To address this, a key strategy will be to form international collaborations for improvement in Annonas, thus facilitating resources and knowledge sharing. Ultimately, efforts should also focus on translating research outputs into tangible applications for growers and breeders.

## Data Availability

No datasets were generated or analysed during the current study.
